# Supplementary material for: Enhancing Antibiotic Efficacy with Natural Compounds: Synergistic Activity of Tannic Acid and Nerol with Commercial Antibiotics against Pathogenic Bacteria
Source: Plants (Basel). 2024 Sep 28;13(19):2717. doi: 10.3390/plants13192717 (PMC11479191; doi:10.3390/plants13192717)
Supplement: Supplementary file 1 [file plants-13-02717-s001.zip › plants-3142681-supplementary.pdf]

Table S1. Antimicrobial activity of antibiotics (MIC, μg/mL) on pathogenic bacteria tested.

Microorganism	PEN	AMP	AMO	GTM	STM	ERY	TC	CHL
Escherichia coli (ATCC 25922)	-	7.80	7.80	12.50	9.37	300	0.78	7.81
Salmonella enterica (ATCC 13311)	-	16.62	62.50	0.78	4.68	37.50	1.17	3.91
Klebsiella pneumoniae (C6)	-	62.50	250	0.78	9.37	18.75	1.17	7.81
Serratia marcescens (ATCC 13880)	-	6.25	125	5	0.40	250	62.50	62.50
Proteus mirabilis (ATCC 35659)	-	0.078	0.62	-	12.50	-	-	3.12
Pseudomona aeruginosa (ATCC 27853)	-	-	949	5	72.25	-	30	>125
Klebsiella aerogenes (ATCC 13048)	-	>250	>949	6.25	1.56	75	1.90	3.91
Acinetobacter baumannii (ATCC 19606)	500	125	125	12.50	75	10	0.78	62.50
Bacillus subtilis (ATCC 6633)	-	-	0.31	6.25	6.25	-	5	1.56
Staphylococcus aureus (ATCC 9144)	1.25	0.15	0.625	50	50	0.62	0.31	7.50
Enterococcus faecalis (ATCC 19433)	>1042	62.50	0.15	25	6.25	6.25	12.50	100
Streptococcus agalactiae (ATCC 12386)	0.156	0.15	16.625	25	125	<0.4	0.19	14
Pasteurella aerogenes (ATCC 27883)	>500	-	>500	15.625	25	>500	3.125	8

- Not tested

PEN, penicillin; AMP (Ampicillin); AMO (Amoxicillin); GTM (Gentamicin); STM (Streptomycin); ERY (Erythromycin); TC (Tetracycline) and CHL (Chloramphenicol).

Table S2. Microorganisms reference and culture conditions according to American Type Culture Collection (ATCC) datasheets for each microorganism.

<table>
<tr><th colspan="7">Microorganism culture conditions</th></tr>
<tr><th>Microorganism</th><th>Reference</th><th>GRAM</th><th>Temperature (ºC)</th><th>Time (h)</th><th>Agar/Broth</th><th>Atmosphere</th></tr>
<tr><td>Bacillus subtilis</td><td>ATCC 6633</td><td rowspan="4">positive</td><td>30</td><td rowspan="6">24</td><td>BHI</td><td rowspan="15">Aerobic</td></tr>
<tr><td>Staphylococcus aureus</td><td>ATCC 9144</td><td rowspan="5">37</td><td>TS</td></tr>
<tr><td>Enterococcus faecalis</td><td>ATCC 19433</td><td rowspan="2">BHI</td></tr>
<tr><td>Streptococcus agalactiae</td><td>ATCC 12386</td></tr>
<tr><td>Escherichia coli</td><td>ATCC 25922</td><td rowspan="10">negative</td><td>TS</td></tr>
<tr><td>Klebsiella pneumoniae</td><td>C6</td><td rowspan="2">NU</td></tr>
<tr><td>Serratia marcescens subsp.marcescens</td><td>ATCC 13880</td><td>26</td><td>24-48</td></tr>
<tr><td>Proteus mirabilis</td><td>ATCC 35659</td><td rowspan="2">37</td><td rowspan="8">24</td><td rowspan="2">TS</td></tr>
<tr><td>Pseudomonas aeruginosa</td><td>ATCC 27853</td></tr>
<tr><td>Klebsiella aerogenes</td><td>ATCC 13048</td><td>30</td><td rowspan="2">NU</td></tr>
<tr><td>Acinetobacter baumannii</td><td>ATCC 19606</td><td rowspan="4">37</td></tr>
<tr><td>Pasteurella aerogenes</td><td>ATCC 27883</td><td>BHI</td></tr>
<tr><td>Salmonella entérica</td><td>ATCC 13311</td><td rowspan="2">NU</td></tr>
<tr><td>Acinetobacter baumannii</td><td>ATCC 19606</td></tr>
<tr><td>Candida albicans</td><td>ATCC 10231</td><td></td><td>25-30</td><td>SDB</td></tr>
<tr><td colspan="7">TS -Trypticase Soy Agar/Broth, NU - Nutrient agar or nutrient broth, BHI - Brain Heart Infusion Agar/Broth, SDB - Sabouraud Dextrose Broth</td></tr>
</table>
